# EMPeror: a tool for visualizing high-throughput microbial community data

**DOI:** 10.1186/2047-217X-2-16

**Published:** 2013-11-26

**Authors:** Yoshiki Vázquez-Baeza, Meg Pirrung, Antonio Gonzalez, Rob Knight

**Affiliations:** 1 Department of Computer Science, University of Colorado at Boulder, Boulder, CO 80309, USA; 2 Department of Pharmacology, University of Colorado Denver, Aurora, CO 80045, USA; 3 BioFrontiers Institute, University of Colorado at Boulder, Boulder, CO 80309, USA; 4 Department of Chemistry & Biochemistry, University of Colorado at Boulder, 80309, Boulder, CO, USA; 5 Howard Hughes Medical Institute, Boulder, CO, 80309, USA

**Keywords:** Microbial ecology, QIIME, Data visualization

## Abstract

**Background:**

As microbial ecologists take advantage of high-throughput sequencing technologies to describe microbial communities across ever-increasing numbers of samples, new analysis tools are required to relate the distribution of microbes among larger numbers of communities, and to use increasingly rich and standards-compliant metadata to understand the biological factors driving these relationships. In particular, the Earth Microbiome Project drives these needs by profiling the genomic content of tens of thousands of samples across multiple environment types.

**Findings:**

Features of EMPeror include: ability to visualize gradients and categorical data, visualize different principal coordinates axes, present the data in the form of parallel coordinates, show taxa as well as environmental samples, dynamically adjust the size and transparency of the spheres representing the communities on a per-category basis, dynamically scale the axes according to the fraction of variance each explains, show, hide or recolor points according to arbitrary metadata including that compliant with the MIxS family of standards developed by the Genomic Standards Consortium, display jackknifed-resampled data to assess statistical confidence in clustering, perform coordinate comparisons (useful for procrustes analysis plots), and greatly reduce loading times and overall memory footprint compared with existing approaches. Additionally, ease of sharing, given EMPeror’s small output file size, enables agile collaboration by allowing users to embed these visualizations via emails or web pages without the need for extra plugins.

**Conclusions:**

Here we present EMPeror, an open source and web browser enabled tool with a versatile command line interface that allows researchers to perform rapid exploratory investigations of 3D visualizations of microbial community data, such as the widely used principal coordinates plots. EMPeror includes a rich set of controllers to modify features as a function of the metadata. By being specifically tailored to the requirements of microbial ecologists, EMPeror thus increases the speed with which insight can be gained from large microbiome datasets.

## Findings

### Background

Rapid increases in sequencing capacity are greatly expanding our ability to understand the microbial world: scaling from a handful of samples to hundreds, or thousands, allows a rich picture of trends over temporal and spatial scales that were previously unattainable. Human microbiome studies are not the only beneficiaries of this ability to perform increased sampling: large-scale patterns are now being discovered in communities ranging from soils [1] to oceans [2] including the efforts from the International Census of Marine Microbes (ICoMM). We can now process thousands of samples in a single sequencing run [3], and in turn computational tools must also scale to fulfill these needs [4].

**Figure 1:**
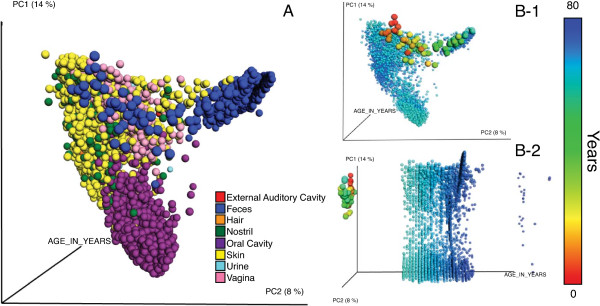
**EMPeror display showing the combination of the datasets described in [**[3]**,**[20]**-**[22]**], consisting of 5740 samples representing human auditory canal, skin, nostril, feces, vagina, urine, hair and oral body habitats. (A)** Data colored by body habitat; **(B-1)** Principal coordinate 1 (PC1) vs. PC2 with the same the data colored according to the age of the subjects (a continuous variable). **(B-2)** PC1 vs. an explicit time axis. The results allow us to see by immediate visual inspection that the body habitats are remarkably different between each other and that this is consistent through time as a human reaches adulthood.

Although data visualization is an empowering tool that allows an efficient understanding of information [5], it remains a major challenge in this area of study, specifically because with more samples comes richer information relating the samples to one another (this contextual information is often referred to as “sequence metadata”) and to the study design itself. When analyzing large numbers of samples, researchers need to know the patterns that link specific samples or microbes to overall patterns of diversity, and to different metadata variables: this is typically critical for usable visualizations. A well know ecological metric to quickly compare the microbial composition of the samples is beta diversity, which collates them by creating a distance matrix of these differences. Ordination methods, such as Principal Coordinates Analysis (PCoA) [6] are useful for dimensionality reduction and widely used in different fields to conceptualize distance matrices, however determining how to visualize the samples to reveal clear patterns often remains a challenge. Figure 1A shows the samples colored by the body site each belong to, a common approach that will make evident the main differences explained in the first two axes of variation; however, when integrating metadata in the coloring patterns (Figure 1B-1, B-2), the plot clearly shows the age differences between the samples of an infant, compared to the samples belonging to healthy human adults.

There are several existing methods for displaying PCoA results, but none to date are specifically designed to account for the common use cases in this research field; furthermore, each of the most representative solutions allots different limitations. For example, QIIME [7], an open source framework for upstream and downstream analysis of microbial community samples generated via high-throughput sequencing instruments, typically generates 3D plots using KiNG [8] originally designed as a molecular graphics viewer, which requires static files containing each metadata field to be produced in advance, replicating the coordinates for each of these categories and resulting in long load times and large file sizes when the metadata are rich. SpotFire [9] is a very expensive commercial solution, beyond the budget of many research laboratories. Generic packages that provide 3D plotting functionalities such as MATLAB [10], Mathematica [11], R [12], Excel [13] or Matplotlib [14] can always be used, but custom code or manual approaches are typically required to relate each point to a specific visual feature intended to highlight a given variable. Consequently, this could become a time-consuming process, which as a side effect compromises its reliability, reusability and reproducibility. Moreover, none of the previously mentioned applications are specifically modeled to support the workflows of the modern microbial ecologist. Allowing the user to choose among metadata coloring dynamically, and separating coloring from visibility, has a surprisingly large effect in encouraging interactive exploration, understanding and analysis, and often allows insights into the main factors, as well as more subtle ones, structuring the data to be obtained much more rapidly.

## EMPeror

EMPeror is a thoroughly tested and open-source software package with an interactive user interface and hardware-accelerated graphics, implemented with HTML5, WebGL, Javascript and Python, and tightly integrated with QIIME [7] and PyCogent [15]. EMPeror’s command line interface accepts QIIME principal coordinates files and metadata mapping files, and produces an interactive 3D visualization that can be delivered in the context of a web page independent of the command line tool. As an example of EMPeror’s ability to deal with continuous variables (time, alpha diversity, pH) that are part of the metadata, these factors can be integrated as an explicit axis in the plot, lines connecting subsequent points of single trajectories (treatments, subjects, sites, etc.) or using a colormap to have each sample’s color be a function of its position in the gradient. The main features that EMPeror provides are: (1) easily change visibility features of data points in the plots based on metadata; (2) can be easily embedded into other tools, such as Evident [16] as a reusable visualization component; (3) scale to thousands of points with minimal load times (seconds versus many minutes in KiNG); and (4) ability to display auxiliary data to increase the understanding of the intrinsic data patterns; these include: biplots [17], procrustes analysis [18], and jackknifed beta diversity plots [19].

To illustrate the effectiveness of EMPeror, we show the combination of [3,20-22], see Table 1, as generated with the QIIME web application [23]. This combination represents 5,740 samples (spheres), and 120 columns of metadata [24]. In KiNG, the resulting files for both the discrete and gradient coloring result in a size of 1.85 GB, but in EMPeror only 26 MB [25], meaning only 1.3% of the original size, see Figure 1. Additionally, we can easily view the intrinsic age patterns within the data, Figure 1B, both panels.

**Table 1:** **Studies used to create Figure**1

**Title**	**General description**	**Collected samples**	**Reference**
Moving pictures of the human microbiome	Samples from two subjects are collected for up to 15 months in three body sites (oral, skin and gut)	1964	[3]
Bacterial community variation in human body habitats across space and time	Samples from healthy adult human samples from eight subjects of up to 27 body sites	585	[20]
Structure, function and diversity of the healthy human microbiome	Samples from 242 healthy adult human samples from up to eighteen different body sites	3131	[21]
Succession of microbial consortia in the developing infant gut microbiome	Gut samples collected biweekly from an infant through the first 2.5 years of life	60	[22]

EMPeror installation instructions can be found in the online documentation (http://qiime.org/emperor/installation_index.html).

## Conclusions

EMPeror provides a user-friendly interface and set of tools for visualizing large numbers of microbial community samples associated with increasingly extensive metadata, and interactively manipulating these datasets to add auxiliary data and visualization techniques. Additionally, it contains several user interface features, enabling straightforward modifications and customization of perceptible aspects in the plot plus the incorporation of statistical techniques, which also help increase the ease and speed of exploratory analysis. We believe that EMPeror will have a large impact on the field, especially for large-scale environmental sampling projects, such as the Earth Microbiome Project [26], and large-scale clinical projects, such as the Human Microbiome Project [20].

## Availability and requirements

**Project name**: Emperor

**Project home page**: http://emperor.colorado.edu

**Operating system(s)**: Platform independent for the graphical user interface; OS X (10.6 and higher) and Linux only for the command line interface.

**Programming language**: Python and JavaScript.

**Other Requirements**: Python 2.7, Chrome, QIIME (python libraries only), NumPy, BIOM 1.1.0 and PyCogent.

**License**: Modified BSD.

**Any restrictions to use by non-academics:** None.

## Availability of supporting data

The example files and additional data sets supporting the results of this article are available in the GigaScience Database [24], as well as from the EMPeror FTP site [25].

## Abbreviations

EMP: Earth Microbiome Project; HTML5: HyperText Markup Language, version 5; ICoMM: International Census of Marine Microbes; MIxS: Minimum information about any (x) sequence; NumPy: Numerical Python; PCoA: Principal Coordinates Analysis; PyCogent: Comparative and Genomic Toolkit; QIIME: Quantitative Insights into Microbial Ecology; WebGL: Web Graphics Library.

## Competing interests

The authors declare that they have no competing interests.

## Authors’ contributions

YVB, MP and AG developed parts of the visualization and backend frameworks for EMPeror. YVB, AG and RK wrote the manuscript. AG, and RK established the initial design and goals of the project. All authors read and approved the final manuscript.

## Authors’ information

YVB, AG, MP and RK are developers and or leaders of the QIIME project.
